# Current treatment options in *RAS* mutant metastatic colorectal cancer patients: a meta-analysis of 14 randomized phase III trials

**DOI:** 10.1007/s00432-020-03290-y

**Published:** 2020-06-19

**Authors:** Arndt Stahler, Volker Heinemann, Ingrid Ricard, Jobst C. von Einem, Clemens Giessen-Jung, Christoph Benedikt Westphalen, Marlies Michl, Kathrin Heinrich, Lisa Miller-Phillips, Ivan Jelas, Sebastian Stintzing, Dominik Paul Modest

**Affiliations:** 1 Department of Medicine III and Comprehensive Cancer Centre, University Hospital Grosshadern, University of Munich, Marchioninistrasse 15, 81377 Munich, Germany; 2 DKTK, German Cancer Consortium, German Cancer Research Centre (DKFZ), Heidelberg, Germany; 3 Department of Hematology, Oncology, and Tumor Immunology (CCM), Charité-Universtiaetsmedizin, Berlin, Germany; 4 Department of Hematology, Oncology, and Tumor Immunology (CVK), Charité-Universtiaetsmedizin, Berlin, Germany

**Keywords:** *RAS*, Angiogenic, Chemotherapy, Metastatic, Colorectal cancer

## Abstract

**Purpose:**

Although biomarkers for patients with metastatic colorectal cancer exist, the benefit patients with *RAS* mutated tumors derive from established regimens is unclear.

**Methods:**

Efficacy of therapeutic strategies available for *RAS* mutated patients (addition of chemotherapeutic agents and/or anti angiogenic agents) were investigated in fourteen randomized controlled phase III trials at trial level by meta-analysing individual study hazard ratios and 95% confidence intervals (95% CI) for overall survival (OS) and progression free survival (PFS).

**Results:**

6810 of 10,748 patients (63.3%) were available (48.5% *RAS* wildtype, 51.5% *RAS* mutated). Across all treatment lines, additional treatment efficacy (chemotherapy and/or anti angiogenic agents) was significantly smaller in *RAS* mutated compared to wildtype tumors for OS and PFS. In detail, patients with *RAS* mutated metastatic colorectal cancer derived significant benefit in PFS but not in OS by the addition of either chemotherapy or anti angiogenic agents to the respective comparator. In patients with *RAS* wildtype metastatic colorectal cancer, PFS and OS were improved by the addition of chemotherapy or anti angiogenic agent.

**Conclusion:**

The therapeutic benefit of additional substances is less distinct in patients with *RAS* mutated as compared to *RAS* wildtype metastatic colorectal cancer, especially with regard to OS.

**Electronic supplementary material:**

The online version of this article (10.1007/s00432-020-03290-y) contains supplementary material, which is available to authorized users.

## Background

The *RAS* protein is a member of the G protein family and involved in signal transduction within the mitogen activated protein kinases (*MAPK*) pathway. Genetic alterations lead to constitutive activation of the *RAS* protein with a high oncogenic potential in metastatic colorectal cancer (mCRC) (Benvenuti et al. 2007; Vogelstein et al. 1988). *RAS* mutations (MUT) are detected in about 50% of all patients (Cunningham et al. 2004; Jonker et al. 2007; Sobrero et al. 2008). Since 2013, *RAS* wildtype (WT) status is required for the use of anti-*EGFR* (epidermal growth factor receptor) agents like cetuximab or panitumumab (Douillard et al. 2013; Heinemann et al. 2014).

As *EGF* receptor inhibition is ineffective because of constitutive oncogenic signalling, (Benvenuti et al. 2007) systemic treatment option in patients with a *RAS* MUT tumor currently include chemotherapy (fluoropyrimidines, irinotecan, oxaliplatin) with or without anti angiogenic agents for two treatment lines, followed by later-line treatment such as trifluridine/tipiracil and regorafenib (Grothey et al. 2013; Kubicka et al. 2013; Van Cutsem et al. 2018). For maintenance strategies following induction treatment, a combination of fluoropyrimidine and bevacizumab is usually recommended (Goey et al. 2017; Hegewisch-Becker et al. 2015; Van Cutsem et al. 2016).

Unlike anti-*EGFR* treatment, predictive biomarkers for the use of cytotoxic and anti angiogenic agents are still missing. A comprehensive efficacy analysis of these treatment strategies in *RAS* MUT tumors is currently not available.

We, therefore, performed a systematic review and meta-analysis of randomized controlled phase III trials with EMA/FDA approved cytotoxic and anti angiogenic agents to evaluate efficacy of the addition of chemotherapeutics and anti angiogenic agents when distinguished for *RAS* status, treatment line and investigated agents.

## Methods

### Trial identification

Our search strategy included trial identification by systematic literature review using the following terms: “metastatic colorectal cancer”, “randomized”, “phase III”, “NOT phase II”, “NOT meta”, “NOT pooled”. First search was performed in February 2019 and last search in November 2019. Only trials with available molecular subgroup analysis regarding *(K)RAS* status (*KRAS* exon 2–4, *NRAS* exon 2–4) were included. Hence, we included randomized controlled phase III trials with available subgroup data for (*K)RAS* status in mCRC evaluating the addition of chemotherapeutic or anti angiogenic treatment to a randomised control arm with FDA/EMA approved agents. As treatment efficacy should be evaluated according to (*K)RAS* status, trials with anti-*EGFR* treatment requiring *(K)RAS* wildtype status (cetuximab, panitumumab) were excluded. Patients with *BRAF* mutations were excluded from this analysis if indicated.

Following trials were identified in Pubmed, EMBASE, Web Of Science and the Cochrane Central Register of Controlled Trials (CENTRAL): TRIBE (Cremolini et al. 2015), AVG2107g (Hurwitz et al. 2009), FOCUS (Richman et al. 2009), ML22011 (Modest et al. 2018), AGITG MAX (Price et al. 2015), ML18147 (Kubicka et al. 2013), RAISE (Obermannova et al. 2016), VELOUR (Wirapati et al. 2017), CORRECT (Grothey et al. 2013), CONCUR (Li et al. 2015), RECOURSE (Van Cutsem et al. 2018), AIOKRK0207 (Hegewisch-Becker et al. 2015), CAIRO3 (Goey et al. 2017), PRODIGE 9 (Aparicio et al. 2018). Data were based on publications and/or poster presentations at congress meetings.

### Trials

TRIBE and ML22011 investigated chemotherapeutic (de-)escalation strategies on bevacizumab based treatment arms in previously untreated mCRC. The MRC FOCUS trial compared 5-fluorouracil monotherapy to the combination regimes irinotecan/5-fluorouracil (IrFU) and oxaliplatin/5-fluorouracil (OxFU) as first-line therapy of mCRC. In AVG2107g and AGITG MAX, bevacizumab was used additionally to chemotherapy in untreated mCRC. All second-line trials (ML18147, RAISE, VELOUR) investigated the role of additional anti angiogenic agents to chemotherapy in previously treated patients with mCRC. CORRECT and CONCUR compared regorafenib vs. placebo treatment in mCRC. The RECOURSE trial compared single-agent chemotherapy with TAS102 to best supportive care. In maintenance, most trials investigated treatment with angiogenic inhibition compared to no treatment (AIOKRK0207: bevacizumab ± fluoropyrimidine vs. no treatment; CAIRO3: capecitabine + bevacizumab; PRODIGE 9: bevacizumab).

### Data items, data collection process and summary measures

Retrospective data (hazard ratio with confidence interval) regarding overall survival (OS) and progression-free survival (PFS) were collected to compare outcome of chemotherapeutic and non-chemotherapeutic treatment addition strategies by *(K)RAS* status in patients with previously untreated and treated mCRC and by treatment lines at trial level. Control arms were used as reference, meaning that hazard ratios smaller than 1 indicated benefit of the addition of the respective drug to the treatment protocol.

### Risk of bias in individual studies

To ascertain the validity of eligible randomized trials, two authors (AS, DPM) determined independently the adequacy of trials regarding phase of trial, presence of molecular subgroup analysis and strategies of additional treatment.

### Risk of bias across studies

Primary tumor sidedness, microsatellite instability and type of cytotoxic treatment (FOLFIRI or FOLFOX) were not considered in this analysis and might have affected the cumulative evidence.

### Planned methods of analysis

Standard error estimates were deduced from the 95% confidence intervals. Meta-analyses and meta-regression analyses based on the log-hazard rate ratios were performed. Random effect meta-regression models were fitted for all trials, for each treatment line (first, second, later and maintenance lines) and treatment addition (chemotherapeutic vs. anti angiogenic therapy). Interaction effect of *RAS* mutation type (*RAS* WT vs. *RAS* MUT tumors) with treatment addition was assessed. Heterogeneity explained by mutation was assessed by a Wald chi-square test. Residual heterogeneity was determined by computing the Cochran’s *Q* test (chi-square test) and the *I*^2^ statistic with its 95% confidence interval. In case of three-armed trials, correlation of 0.5 was added to treatment effects to integrate repeated comparisons of the control group to different experimental treatment arms into results. Data analysis was structured to resolve complexity of different result layers. In a first step, benefit of therapeutic addition vs. control was investigated for all patients regardless of therapy strategy or molecular subgroups. Subsequent analyses across all treatment lines were performed separately for *RAS* WT patients and for *RAS* MUT patients, and then for *RAS* WT vs. MUT patients, respectively. Within the molecular subgroups, we first compared efficacy of therapeutic addition vs. control regardless of substance classes. Then, benefit of chemotherapeutic and anti angiogenic strategies were analyzed in detail. Finally, each treatment line including maintenance was stratified by *RAS* WT, *RAS* MUT and *RAS* WT vs. MUT and analyzed for treatment efficacy. Weight of the trials was respected by number of trial patients. All tests were two-sided and the significance level was set to 0.05. The analyses were performed using R 3.6.1, particularly packages *forestplot* and *metafor*.

## Results

### Study selection

**Fig. 1:**
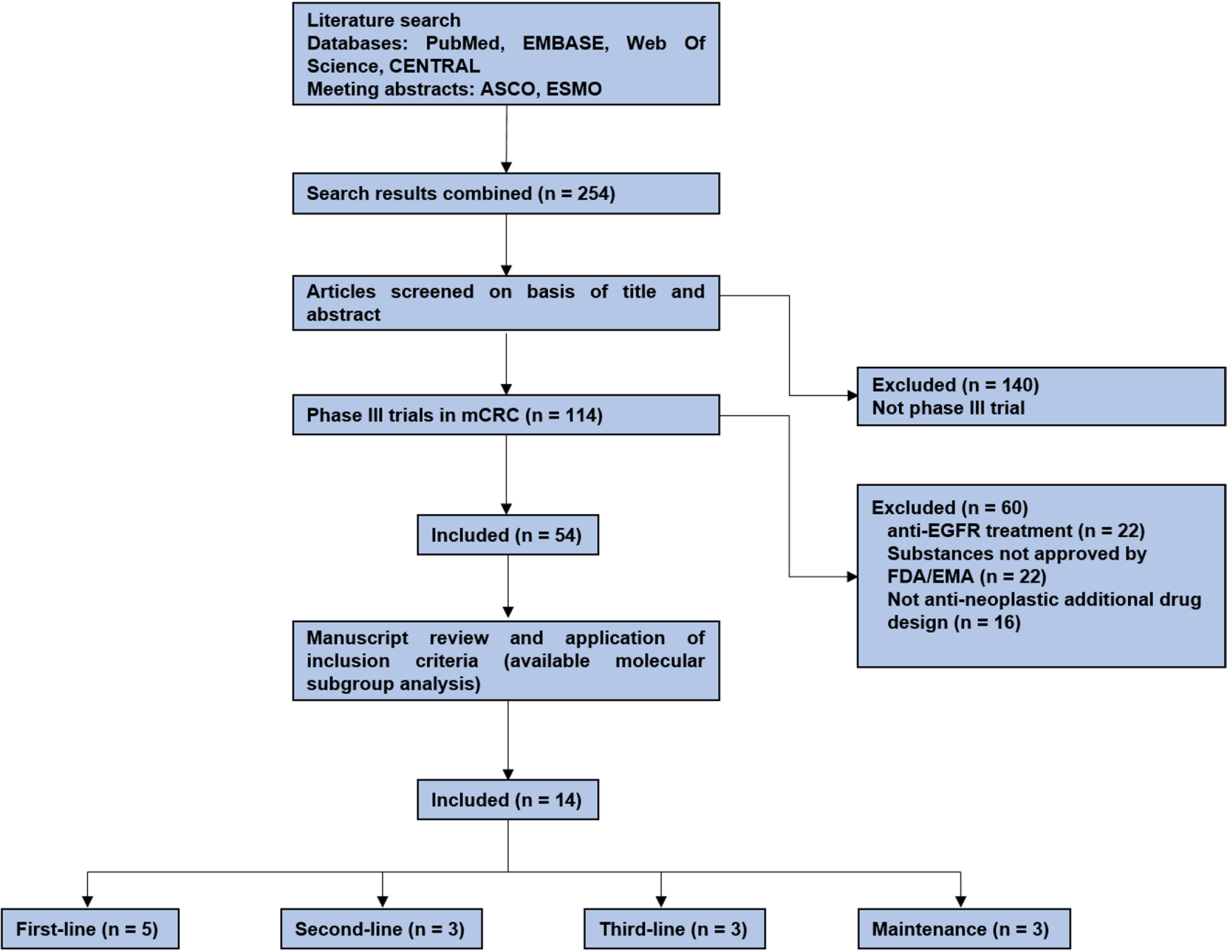
Workflow of trial identification process; *ASCO* American Society of Clinical Oncology, *ESMO* European Society of Medical Oncology

Search terms identified 114 phase III trials in total, of which 60 trials had to be excluded due to anti *EGFR* treatment (22 trials), testing of substances not approved by FDA/EMA for treatment of metastatic colorectal cancer (22 trials) and trial designs which did not compare an additional anti-neoplastic drug to standard treatment (16 trials). Of these remaining trials, 40 trials did not provide molecular subgroup analyses for *(K)RAS* status. (Fig. 1).

### Patients

Fourteen trials comprising 10,748 patients were included into the analysis. 6 810 patients (63.3%) were evaluated according to molecular status. (Table 1) Detailed outcome results for each trial in each treatment line according to *RAS* status were presented in supplementary data. (Online resources 1–4).

**Table 1:** Trial characteristics of the analyzed randomized controlled trials

Trial name	Trial characteristics								
	Phase	Treatment line	Control arm	Escalation arm(s)	Target	Randomized	Tumors collected	With RAS wildtype	With RAS mutated
TRIBE	III	First line	FOLFIRI + Bevacizumab	FOLFOXIRI + Bevacizumab	Chemotherapy	508	391	93	236
AVG2107g	III	First line	IFL	IFL + Bevacizumab	Anti angiogenic	813	230	152	78
FOCUS	III	First line	5-FU	IrFU; OxFU	Chemotherapy	2 135	711	389	300
ML22011	III	First line	FP + Bevacizumab + Irinotecan	FP + Bevacizumab followed by Irinotecan	Chemotherapy	421	374	158	194
AGITG-MAX	III	First line	Capecitabine	Capecitabine + Bevacizumab(+ Mitomycin)	Anti angiogenic	471	280	171	109
ML18147	III	Second line	Chemotherapy	Chemotherapy + Bevacizumab	Anti angiogenic	820	616	316	300
RAISE	III	Second line	FOLFIRI	FOLFIRI + Ramucirumab	Anti angiogenic	1 076	1 072	542	530
VELOUR	III	Second line	FOLFIRI	FOLFIRI + Aflibercept	Anti angiogenic	1 226	482	218	264
CORRECT	III	Later line	Placebo	Regorafenib	Anti angiogenic	753	729	299	430
CONCUR	III	Later line	Placebo	Regorafenib	Anti angiogenic	204	143	79	64
RECOURSE	III	Later line	Best supportive care	Best supportive care + TAS102	Chemotherapy	800	800	393	407
AIOKRK0207	III	Maintenance	No treatment	Bevacizumab	Anti angiogenic	472	335	141	172
CAIRO3	III	Maintenance	No treatment	Capecitabine + Bevacizumab	Chemotherapy	558	420	140	240
PRODIGE 9	III	Maintenance	No treatment	Bevacizumab	Anti angiogenic	491	375	202	173

*RAS* rat sarcoma, *FOLFIRI* 5-fluorouracil/folinic acid/irinotecan, *FOLFOXIRI* 5-fluorouracil/folinic acid/oxaliplatin/irinotecan, *IFL* irinotecan/5-fluorouracil/folinic acid, *VEGF* vascular endothelial growth factor, *5-FU* 5-fluorouracil, *IrFU* irinotecan/5-fluorouracil, *OxFU* oxaliplatin/5-fluorouracil, *FP* fluoropyrimidine, *TAS102* trifluridin/tipiracil, *EGFR* epidermal growth factor receptor, *ctDNA* circulating tumor DNA

### Effect of additional treatment agent (chemotherapy and/or anti angiogenic agent)

Across all trials the benefit in overall survival (OS) (HR 0.83 (95% CI 0.78–0.89), *p* < 0.0001, *p* for heterogeneity = 0.25) and PFS (HR: 0.60 (95% CI 0.54–0.67), *p* < 0.0001, *p* for heterogeneity < 0.0001) was significant.

### Efficacy analysis in *RAS* WT vs. MUT tumors across all treatment lines

The benefit in OS with the addition of chemotherapeutic and/or anti angiogenic agents was significantly greater in *RAS* WT tumors as compared to *RAS* MUT tumors when all studies were analysed together (*p* for interaction = 0.003). In detail, the effect of the addition of a chemotherapeutic agent was less pronounced in patients with RAS MUT mCRC (WT: HR = 0.74, 95%CI 0.64–0.87; MUT: HR = 0.89, 95% CI 0.78–1.02), *p* for interaction = 0.07) and the addition of anti angiogenic treatment was significantly less efficient in *RAS* MUT compared to WT tumors. Interaction of anti angiogenic treatment and *RAS* status was significant (WT: HR = 0.78, 95% CI 0.70–0.87; MUT: HR = 0.91, 95%CI 0.82–1.01; *p* for interaction = 0.039).

**Fig. 2:**
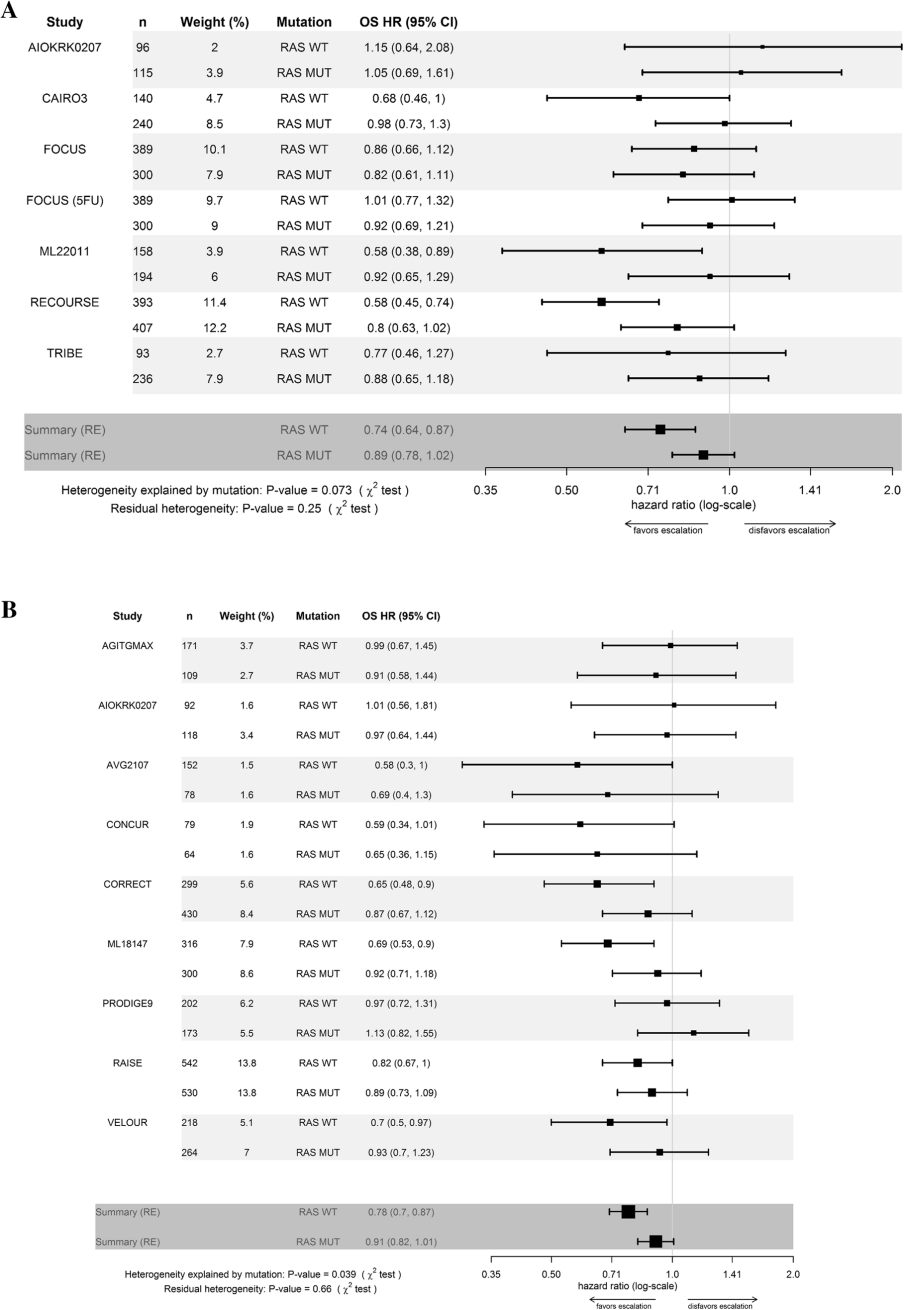
**a** Forest plot of overall treatment effect of chemotherapeutic escalation regarding overall survival for patients with RAS wildtype (WT) and mutated (MUT) tumors **b** Forest plot of overall treatment effect of escalation by anti angiogenic escalation regarding overall survival for patients with RAS wildtype (WT) and (MUT) tumors. *OS* overall survival, *HR* hazard ratio, 95% *CI* 95% confidence interval, *RE* random effects model. FOCUS upfront IrFU/OxFU vs. FU. FOCUS 5-FU: sequential 5-FU then IrFU/OxFU vs. FU

**Fig. 3:**
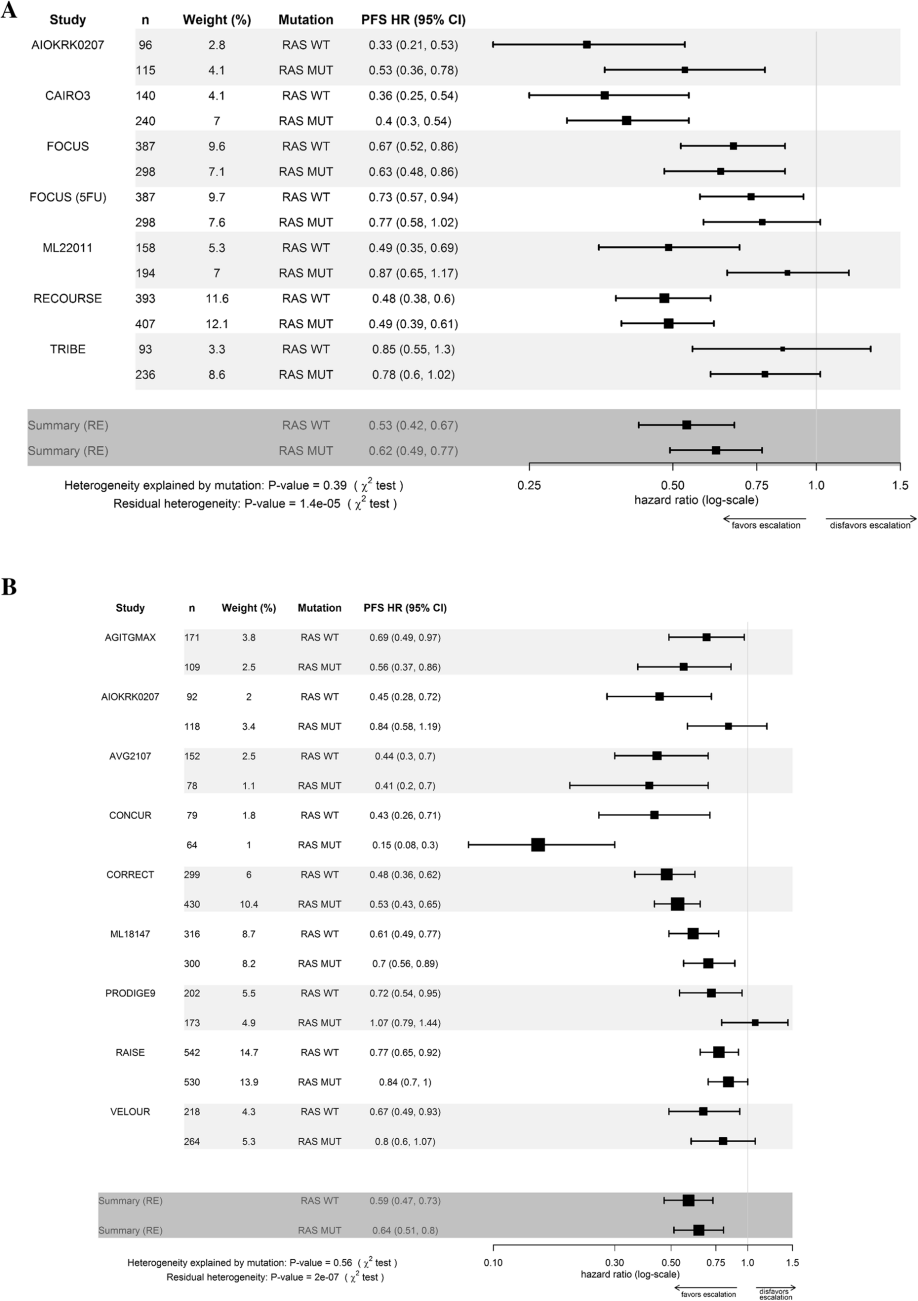
**a** Forest plot of overall treatment effect of chemotherapeutic escalation regarding progression free survival for patients with RAS wildtype (WT) and mutated (MUT) tumors **b** Forest plot of overall treatment effect of escalation by anti angiogenic escalation regarding progression free survival for patients with RAS wildtype (WT) and mutated (MUT) tumors. *PFS* progression-free survival, *HR* hazard ratio, *95% CI* 95% confidence interval, *RE* random effects model. FOCUS: upfront IrFU/OxFU vs. FU. FOCUS 5-FU: sequential 5-FU then IrFU/OxFU vs. FU

Regarding PFS, the effect of addition of chemotherapeutic and/or anti angiogenic agents was comparable in patients with *RAS* WT and MUT tumors.. However, heterogeneity was significant when analysing all trials and the subsets of additional chemotherapeutic or anti angiogenic agents (*p* < 0.0001).]. (Figs. 2, 3, Table 2).

**Table 2:** Efficacy of escalation vs. non-escalation and escalation strategies for OS and PFS (adjusted for trial effect)

Parameter	Therapeutic escalation vs. non-escalation		Therapeutic escalation strategy			
	All escalation strategies		Chemotherapy		Anti angiogenic	
	RAS WT	RAS MUT	RAS WT	RAS MUT	RAS WT	RAS MUT
OS						
HR (95% CI) [*p*-value]	0.74 (0.68–0.82) [< 0.0001]	0.89 (0.81–0.97) [0.007]	0.74 (0.64–0.87) [0.0001]	0.89 (0.78–1.02) [0.098]	0.78 (0.70–0.87) [< 0.0001]	0.91 (0.82–1.01) [0.07]
log(HR)	− 0.298	− 0.12	− 0.29	− 0.11	− 0.25	− 0.095
log(HR_MUT_)—log(HR_WT_)	0.178		0.183		0.157	
*p* value for interaction	0.003		0.07		0.039	
*p* value for heterogenity	0.93		0.25		0.66	
PFS						
HR (95% CI) [*p*-value]	0.55 (0.50–0.61) [< 0.0001]	0.61 (0.56–0.68) [< 0.0001]	0.53 (0.42–0.67) [< 0.0001]	0.62 (0.49–0.77) [< 0.001]	0.59 (0.47–0.73) [< 0.0001]	0.64 (0.51–0.80) [< 0.0001]
log(HR)	− 0.597	− 0.487	− 0.63	− 0.48	− 0.54	− 0.443
log(HR_MUT_)—log(HR_WT_)	0.111		0.142		0.093	
*p* value for interaction	0.093		0.39		0.56	
*p* value for residual heterogenity	0.029		< 0.0001		< 0.0001	

*RAS* rat sarcoma, *WT* wildtype, *MUT* mutated, *OS* overall survival, *PFS* progression free survival, *log HR* natural logarithm of hazard ratio, *VEGF* vascular endothelial growth factor

### Efficacy according to *RAS* WT or MUT tumors for each treatment line

OS was improved regardless of treatment line in *RAS* WT patients. In patients with *RAS* MUT mCRC, the relative improvement of additional treatments was greater in first and later-line treatment, while patients in second-line (*p* for interaction = 0.07) did not benefit from additional therapy. (Table 3) PFS was improved with the addition of agents in all treatment lines.

**Table 3:** Efficacy of therapeutic escalation in each treatment line in RAS WT vs. mut tumors

Parameter	Treatment lines							
	First line		Second line		Later line		Maintenance	
	RAS WT	RAS MUT	RAS WT	RAS MUT	RAS WT	RAS MUT	RAS WT	RAS MUT
OS								
HR (95% CI) [*p*-value]	0.83 (0.71–0.98) [0.026]	0.87 (0.75–1.02) [0.08]	0.76 (0.66–0.87) [0.0001]	0.91 (0.79–1.04) [0.17]	0.60 (0.50–0.73) [< 0.0001]	0.81 (0.69–0.96) [0.017]	0.89 (0.71–1.10) [0.27]	1.03 (0.86–1.24) [0.72]
log(HR)	− 0.19	− 0.13	− 0.28	− 0.096	− 0.50	− 0.20	− 0.12	0.03
log(HR_MUT_)—log(HR_WT_)	0.052		0.18		0.30		0.155	
*p* value for interaction	0.65		0.072		0.018		0.28	
*p* value for heterogenity	0.57		0.85		0.88		0.75	
PFS								
HR (95% CI) [*p*-value]	0.64 (0.55–0.73) [< 0.0001]	0.72 (0.63–0.83) [< 0.0001]	0.70 (0.60–0.80) [< 0.0001]	0.78 (0.68–0.91) [0.0009]	0.47 (0.30–0.72) [0.0005]	0.39 (0.25–0.61) [< 0.0001]	0.60 (0.46–0.78) [< 0.0001]	0.87 (0.69–1.10) [< 0.0001]
log(HR)	− 0.45	− 0.33	− 0.36	− 0.242	− 0.76	− 0.94	− 0.66	− 0.42
log(HR_MUT_)—log(HR_WT_)	0.126		0.12		0.18		0.249	
*p* value for interaction	0.22		0.24		0.57		0.066	
*p* value for residual heterogenity	0.08		0.38		0.01		< 0.0001	

*RAS* rat sarcoma, *WT* wildtype, *MUT* mutated, *OS* overall survival, *PFS* progression free survival, *log HR* natural logarithm of hazard ratio

Maintenance options did not improve OS, but PFS with a trend towards higher efficacy in patients with *RAS* WT compared to MUT tumors (*p* for interaction = 0.066) (Table 3).

## Discussion

Our analysis was motivated by the limited evidence regarding the benefit of adding further treatment to standards (control arms) in *RAS* MUT mCRC. One prior meta-analysis focussed on the benefit of the addition of bevacizumab to first-line treatment and found significantly prolonged PFS but not OS in currently used treatment regimen containing infusional 5-fluoruracil and irinotecan. However, molecular subgroups were not analysed. (Baraniskin et al. 2019) Therefore, we analysed data from fourteen randomized controlled phase III trials with available molecular subgroup data for *RAS* testing in mCRC across several treatment lines.

Mutations in *KRAS* and *NRAS* genes constitutively activate the *RAS* G protein with a high oncogenic potential in the *MAPK* signaling pathway. (Benvenuti et al. 2007) Thus, *RAS* mutations were often associated with worse prognosis of mCRC—both due to different biology and due to lack of anti-*EGFR* targeted therapy. (Andreyev et al. 2001, 1998; Barault et al. 2008; Cremolini et al. 2015; Hegewisch-Becker et al. 2018; Modest et al. 2016; Richman et al. 2009).

Generally, the addition of chemotherapeutic and/or anti angiogenic agents demonstrated a significant benefit in patients *RAS* WT and MUT tumors in our meta analysis in terms of OS and PFS. However, in patients with *RAS* MUT tumors the benefit in OS with the addition of a new agent across all trials and treatment lines was a modest relative risk reduction for death of 12%. Although statistically significant, it might be argued if 12% can be regarded as clinically meaningful improvement. Overall, the addition of agents to the comparators was significantly more effective in patients with *RAS* WT tumors when compared to *RAS* MUT tumors in OS and PFS (see Table 2). This finding may suggest that *RAS* WT mCRC represents a more treatment sensitive entity in as compared to *RAS* MUT mCRC independently from anti-*EGFR* antibody therapy.

When studies investigating chemotherapeutic agents were analysed separately, a trend towards limited efficacy was observed in *RAS* MUT tumors for OS, but not for PFS. The relative risk reduction in *RAS* MUT tumors in this respective setting was only 11% for OS compared to 26% in patients with *RAS* WT mCRC. Importantly, OS benefit from anti angiogenic treatment was significantly smaller in patients with *RAS* MUT tumors as compared to *RAS* WT tumors (see Table 2). These results overlap with our findings of less meaningful benefit in second-line treatment, as included second-line trials investigated anti angiogenic treatment only. Overactivation of the *MAPK* signalling pathway was shown to stimulate angiogenesis *VEGF*-independently and might be a reason for low efficacy of anti angiogenic treatment in patients with *RAS* MUT tumors. (Mehta and Besner 2007).

With a detailed view on different treatment lines, later-line treatment (as compared to control) improved OS to a greater extent in patients with *RAS* WT compared to patients with *RAS* MUT tumors. Although a certain benefit of later-line therapy was also observed *RAS* MUT mCRC, the hazard ratio for OS was only 0.81 (see Table 3). This limited efficacy in this treatment setting needs to be considered carefully in the context of short observation time (absolute benefit is very moderate) and resulting adverse effects and their impact on quality of life in end-stage cancer patients.

Concerning maintenance therapy, our meta-analysis included only trials that compared bevacizumab or capecitabine plus bevacizumab to best supportive care (BSC). (Aparicio et al. 2018; Goey et al. 2017; Hegewisch-Becker et al. 2015) A significant effect on overall survival was seen in neither *RAS* WT nor *RAS* MUT patients, while PFS trended to be improved in *RAS* WT mCRC. When stratified by substances, addition of anti angiogenic therapy alone did not improve outcome in the maintenance setting, while the combination of capecitabine and bevacizumab improved OS in patients with *RAS* wildtype tumors. These results might again strengthen the hypothesis of limited benefit of anti angiogenic therapy in patients with *RAS* MUT mCRC. Therefore, our findings raise the question if maintenance strategies (instead of treatment holidays) should be promoted in patients with *RAS* MUT tumors. Compared to active therapy, careful observation may provide a more quality of life friendly approach without significant impairment of outcome in patients with *RAS* MUT mCRC.

With 6 810 patients, our meta- analysis represents the one of the largest analyses in this setting so far and only randomized trials investigating FDA approved drugs were included. However, several limitations need to be mentioned. As no individual patient data were available, published hazard ratios and confidence intervals had to be obtained from data extraction. Additionally, two trials contained old treatment regimen (IFL and IrFU/OxFU, respectively) that are not recommended anymore (Hurwitz et al. 2009; Richman et al. 2009). Our treatment subgroups contained more anti angiogenic*-*based studies than chemotherapy investigating trials. In particular, data for studies with chemotherapeutic agents beyond first-line therapy are rare (only one further line trial) (Van Cutsem et al. 2018). This clear relation of treatment lines and substance classes might have biased our observation. In AIOKRK0207, outcome was distinguished between between double wildtype mutational status and any mutation only. Therefore, patients with *BRAF* MUT tumors might have biased AIOKRK0207 results in our analysis, although the number should be limited. As most of our investigated trials did not distinguish for primary tumor side and microsatellite (in)stability, we were not able to conduct side-related subgroup analyses. Furthermore, potential treatment interaction might have occurred, since irinotecan and oxaliplatin were used for cytotoxic treatment. Lastly, significant heterogeneity was observed for PFS evaluation in some sub-analyses.

## Summary

In summary, our meta-analyses suggests that the addition of chemotherapeutic and/or anti angiogenic agents optimizes outcome in *RAS* WT, but not necessarily in *RAS* MUT mCRC. Treatment efficacy in *RAS* MUT compared to WT mCRC was significantly less evident with advancing treatment lines. Furthermore, in this analysis, maintenance options improved neither OS nor PFS in patients with *RAS* MUT tumors. Although anti angiogenic therapy is available irrespective of *RAS* status, our overall analysis demonstrates meaningful efficacy predominantly in *RAS* wildtype mCRC.

## Electronic supplementary material

Below is the link to the electronic supplementary material.

Supplementary file1 (PDF 257 kb)

## Data Availability

All data and material are held by the authors’ institution and may be available upon request.
